# Temperature and Drug Treatments in Mevalonate Kinase Deficiency: An *Ex Vivo* Study

**DOI:** 10.1155/2013/715465

**Published:** 2013-09-01

**Authors:** Paola Maura Tricarico, Giulio Kleiner, Elisa Piscianz, Valentina Zanin, Lorenzo Monasta, Sergio Crovella, Annalisa Marcuzzi

**Affiliations:** ^1^Department of Medical, Surgical and Health Sciences, University of Trieste, 34127 Trieste, Italy; ^2^Institute for Maternal and Child Health, IRCCS “Burlo Garofolo,” Via dell'Istria 65/1, 34137 Trieste, Italy

## Abstract

Mevalonate Kinase Deficiency (MKD) is a rare autosomal recessive inborn disorder of cholesterol biosynthesis caused by mutations in the mevalonate kinase (MK) gene, leading to MK enzyme decreased activity. The consequent shortage of mevalonate-derived isoprenoid compounds results in an inflammatory phenotype, caused by the activation of the NALP3 inflammasome that determines an increased caspase-1 activation and IL-1**β** release. In MKD, febrile temperature can further decrease the residual MK activity, leading to mevalonate pathway modulation and to possible disease worsening. We previously demonstrated that the administration of exogenous isoprenoids such as geraniol or the modulation of the enzymatic pathway with drugs, such as Tipifarnib, partially rescues the inflammatory phenotype associated with the defective mevalonic pathway. However, it has not been investigated yet how temperature can affect the success of these treatments. Thus, we investigated the effect of temperature on primary human monocytes from MKD patients. Furthermore the ability of geraniol and Tipifarnib to reduce the abnormal inflammatory response, already described at physiological temperature in MKD, was studied in a febrile condition. We evidenced the role of temperature in the modulation of the inflammatory events and suggested strongly considering this variable in future researches aimed at finding a treatment for MKD.

## 1. Introduction

Mevalonate Kinase Deficiency (MKD), a rare autoinflammatory disease (OMIM no. 251170), is caused by mutations in the second enzyme of the mevalonate pathway (mevalonate kinase (MK)) resulting in reduced enzymatic activity and in the consequent shortage of downstream compounds [1].

MKD has an early onset usually in infancy and affects both sexes equally. A delay in molecular diagnosis frequently occurs, and systemic reactive AA amyloidosis, a form of amyloidosis, may be a severe long-term complication of this condition [2–4]. Different degrees of MKD severity were observed depending on MK residual activity, ranging from an autoinflammatory phenotype characterized by recurrent inflammatory episodes (Hyper-IgD Syndrome (HIDS), OMIM no. 260920) to a more severe clinical presentation, including neurological and psychomotor impairment (mevalonic aciduria (MA), OMIM no. 610377).

The lack of the mevalonate intermediate compound geranylgeranyl pyrophosphate, resulting in the increased caspase-1 activation and IL-1*β* release, has been recently reported as the main pathogenic mechanism in MKD [5, 6].

Multiprotein complexes called inflammasomes are capable of activating caspase-1, in response to many types of stimuli, including microbial and danger/stress [7]. In particular, NALP3 inflammasome seems to be involved in the pathogenesis of MKD: inflammasome activation causes the increased secretion of IL-1*β* [8] and the pyroptosis or caspase-1 dependent cell death [9].

The main phenotypic characteristic of MKD patients is periodic fever. Temperature, indeed, plays a role in MKD, as elevated temperature (40°C) can reduce even more the enzymatic activity of mutated MK, increasing the inflammatory response. However, an increase in HMG-CoA reductase activity will occur afterwards and compensate for the defect, allowing the resolution of the fever attack. These observations permitted to hypothesize that an increase in temperature may be involved in triggering the attacks [10].

Although in the last decade the knowledge of MKD pathogenesis has increased, an etiologic treatment for MKD is still unavailable, and anti-inflammatory drugs [11] as well as novel biologic treatments [12, 13] are currently used with different and debatable results.

We recently showed that plant isoprenoids (such as geraniol) [14] and inhibitors of farnesylation (such as Tipifarnib) [15] could reverse the inflammatory response in cellular and animal MKD models [16].

**Figure 1:**
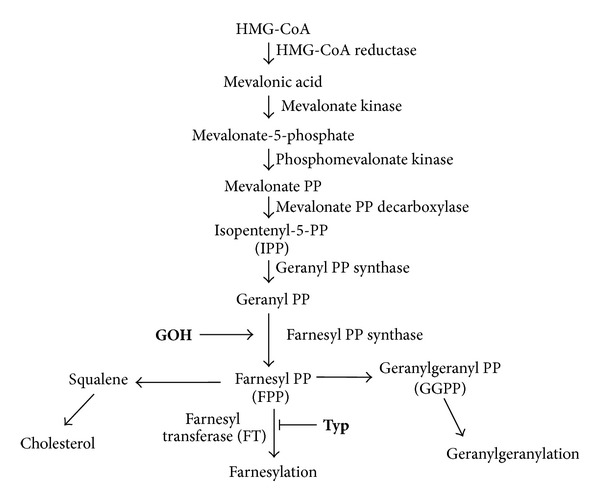
Schematic representation of the mevalonate pathway. Compounds used in the experiments are indicated along the pathway in bold characters: **GOH**: geraniol; **Typ**: Tipifarnib.

Exogenous isoprenoids are able to enter the mevalonate pathway, to be metabolized by the farnesyl pyrophosphate synthase, an enzyme of the mevalonate pathway downstream MK, and to rescue the shortage of intermediate isoprenoids in MKD, models [16]. On the other hand, farnesylation inhibitors can reduce the consumption of farnesyl pyrophosphate, augmenting the geranylgeranyl pyrophosphate available for the geranylgeranylation pathway [17, 18] (Figure 1).

We previously suggested that plant isoprenoids [14] and inhibitors of farnesylation [19] could represent a potential specific pharmacologic approach for MKD; however we did not yet consider how the temperature could act on the anti-inflammatory effect of these compounds in MKD.

To investigate the hypothesis that temperature could affect anti-inflammatory effects, geraniol and Tipifarnib were evaluated in primary human monocytes obtained from MKD patients at different temperatures (37°C and 40°C).

## 2. Materials and Methods

### 2.1. Reagents

Lipopolysaccharide (LPS) (*E*. *coli* serotype 055:B5) and geraniol (GOH) (Sigma-Aldrich, Milano, Italy) were dissolved in saline solution. Tipifarnib ((Typ) R115777, Zarnestra) was dissolved in dimethylsulfoxide (DMSO) so that the final concentration of DMSO would not exceed 0.1%. Tipifarnib was kindly provided by Professor G. Martinelli (Institute of Hematology “L and A Seràgnoli,” University of Bologna, Bologna, Italy).

### 2.2. Subjects

The study was approved by the technical and scientific review board of the Institute for Maternal and Child Health, IRCCS “Burlo Garofolo,” Trieste (no. 185/08, 19/08/2008). For a child to be eligible, informed consent had to be obtained from parents or caregivers. Furthermore, MKD patients of any age were excluded from the study if they had an acute or chronic infectious disease, any clinically significant disorder, and if they were on any medication with known influence on immunological factors (e.g., corticosteroids).

Blood was collected by venipuncture from 3 MKD patients aged between 9 and 12 years (Table 1). MKD patients had no concurrent infection and were not in the acute phase of the disease. Patients' histories of previous infectious diseases and allergy were documented but not evaluated as covariates in the study, because it may affect the results.

### 2.3. Monocyte Isolation of Human Peripheral Blood Monocytes

Monocytes were isolated from MKD patients. Cells were cultured at 2 × 10^5^ cells/well in RPMI 1640 containing 10% FBS (Euroclone, Milan, Italy) with 1 *μ*g/mL LPS for 24 h in presence or not of 100 *μ*M GOH or 5 *μ*M Typ. The same experimental design was performed at 37°C (physiological condition) and at 40°C (acute inflammation condition). At the end of the incubation period the cell culture supernatants were collected for cytokine and inflammasome evaluation while the cells were pelleted for the programmed cell death (PCD) assays.

### 2.4. Programmed Cell Death (PCD) Assays

The PCD of human monocyte was analyzed by flow cytometry using Annexin V (A) and Propidium Iodide (PI) stainings. Cells were stained with FITC-conjugated Annexin V and Propidium Iodide (Annexin V-FITC Apoptosis Detection Kit, Immunostep, Spain) following the manufacturer's indications. In brief, cells were harvested from the culture flasks and washed with PBS. 5 × 10^5^ cells were resuspended in manufacturer buffer and stained with A and PI for 15 minutes. Fluorescence was acquired with a FACScan Cytometer (Becton Dickinson, NJ, USA) and a CellQuest software (Becton Dickinson, NJ, USA) and subsequently analyzed with a FlowJo software (version 7.6, Treestar, Inc., OR, USA). Debris as excluded from the plot based on the scatter; then apoptotic and necrotic cells were characterized based on the fluorescence emitted.

### 2.5. Determination of NALP3 (NLRP3) Concentration

Human NLRP3 concentration was determined in the cell culture medium by enzyme linked immunosorbent assay kits according to manufacturer's protocols, and the amount of NLRP3 was expressed as ng/mL (Human NACHT, LRR and PYD domains-containing protein 3 ELISA Kit; Cusabio Biotech Co., ltd. Carlsbad, CA, USA).

### 2.6. Determination of Cytokines Release

The analysis of a 48 cytokines and chemokines panel (including IL-1*β*, IL-1ra, IL-2, IL-4, IL-5, IL-6, IL-7, IL-8, IL-9, IL-10, IL-12(p70), IL-13, IL-15, IL-17, Eotaxin, FGF basic, G-CSF, GM-CSF, IFN-*γ*, IP-10, MCP-1, MIP-1*α*, PDGF-BB, MIP-1*β*, RANTES, TNF-*β*, VEGF, IL-1*α*, IL-2R*α*, IL-3, IL-12(p40), IL-16, IL-18, CTACK, GRO-*α*, HGF, IFN-*α*2, LIF, MCP-3, M-CSF, MIF, MIG, *β*-NGF, SCF, SCGF-*β*, SDF-1*α*, TNF-*α*, and TRAIL) was performed on supernatant samples using a magnetic bead-based multiplex immunoassays (Bio-Plex) (BIO-RAD Laboratories, Milano, Italy) following manufacturer's instructions. Data from the reactions were acquired using the Bio-Plex 200 reader, while a digital processor managed data output and the Bio-Plex Manager software returned data as median fluorescence intensity (MFI) and concentration (pg/mL).

### 2.7. Data Analysis

For each set of experiments, values were analyzed by calculating means ± standard error (SEM). The nonparametric one-way ANOVA test followed by Bonferroni's correction for multiple comparison test as used when appropriate. Probability (*P*) values were calculated on the basis of one-tailed test. Analysis was performed using GraphPad Prism software version 5.0 (GraphPad Software, Inc., La Jolla, CA, USA). A *P* value of less than 0.05 was considered as statistically significant, unless established by the correction for multiple comparisons.

## 3. Results

Monocytes have been isolated from blood samples of three different MKD patients (Table 1) and tested both at physiological (37°C) and pathological (40°C) temperatures in untreated conditions or after administration of proinflammatory stimulus alone or combined with anti-inflammatory compounds.

### 3.1. Cytokines' Levels in Untreated Conditions or after LPS Administration at 37°C and 40°C

IL-5, IL-7, IL-8, IL-10, IL-13, G-CSF, GM-CSF, MCP-1, MIP-1*β*, RANTES, TNF-*α*, IL-1*α*, IL-2R*α*, IL-3, IL-12(p40), IL-16, IL-18, CTACK, GRO-*α*, HGF, IFN-*α*2, LIF, MCP-3, M-CSF, MIF, MIG, *β*-NGF, SCF, SDF-1*α*, TNF-*β*, and TRAIL did not show any significant difference in untreated conditions nor after the administration of the proinflammatory compound LPS, at neither physiological nor pathological temperatures (Table 2).

**Figure 2:**
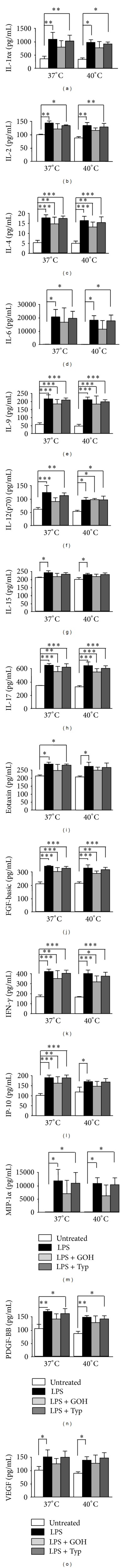
Columns of supernatant level (pg/mL) of IL-1ra, IL-2, IL-4, IL-6, IL-9, IL-12(p70), IL-15, IL-17, Eotaxin, FGF-basic, IFN-*γ*, IP-10, MIP-1*α*, PDGF-BB, and VEGF both at 37°C and at 40°C, in untreated condition and after administration of LPS alone or in combination with GOH or Typ. *P < 0.05; **P < 0.01; ***P < 0.001; one-way ANOVA test followed by Bonferroni's multiple comparison test. LPS: lipopolysaccharide; GOH: geraniol; Typ: Tipifarnib.

On the other hand, levels of IL-1ra, IL-2, IL-4, IL-6, IL-9, IL-12(p70), IL-15, IL-17, Eotaxin, FGF-basic, IFN-*γ*, IP-10, MIP-1*α*, PDGF-BB, and VEGF were significantly dysregulated after the proinflammatory stimulus at 37°C as well as at 40°C. Still, same treatments at different temperatures did not differ significantly (Table 2, Figure 2).

### 3.2. Cytokines' Levels after Administration of Anti-Inflammatory Compounds, GOH and Typ, on LPS-Stimulated Monocytes, at 37°C and 40°C

Levels of cytokines, found to be dysregulated after LPS treatment, have then been evaluated, at both temperatures, after the combined administration of LPS + GOH or LPS + Typ. Drug treatments were not able to significantly nullify the effects of LPS. Nevertheless, a homogeneous trend was observed: GOH better rescued cytokines' levels with respect to Typ in all the tested samples (Table 3, Figure 2).

### 3.3. Temperature-Induced Changes in IL-1*β* Levels

LPS was also able to dysregulate IL-1*β* levels both at 37°C and at 40°C, and it also did so for IL-1ra, IL-2, IL-4, IL-6, IL-9, IL-12(p70), IL-15, IL-17, Eotaxin, FGF-basic, IFN-*γ*, IP-10, MIP-1*α*, PDGF-BB, and VEGF. Even in this case, the combined treatments of LPS + GOH or LPS + Typ did not significantly modify the IL-1*β* levels with respect to the LPS treatment.

**Figure 3:**
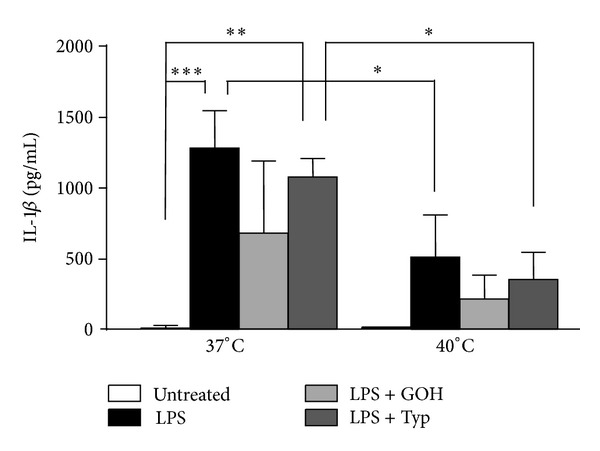
Columns of supernatant level (pg/mL) of IL-1*β*, both at 37°C and at 40°C, in untreated condition and after administration of LPS alone or in combination with GOH or Typ. *P < 0.05; **P < 0.01; ***P < 0.001; one-way ANOVA test followed by Bonferroni's multiple comparison test. LPS: lipopolysaccharide; GOH: geraniol; Typ: Tipifarnib.

Interestingly, IL-1*β* levels at 40°C were notably lower in all experimental conditions with respect to those evaluated at 37°C (Figure 3).

### 3.4. NAPL3 Inflammasome Levels Followed a Temperature Dependent Trend after Inflammatory Stimulus

**Figure 4:**
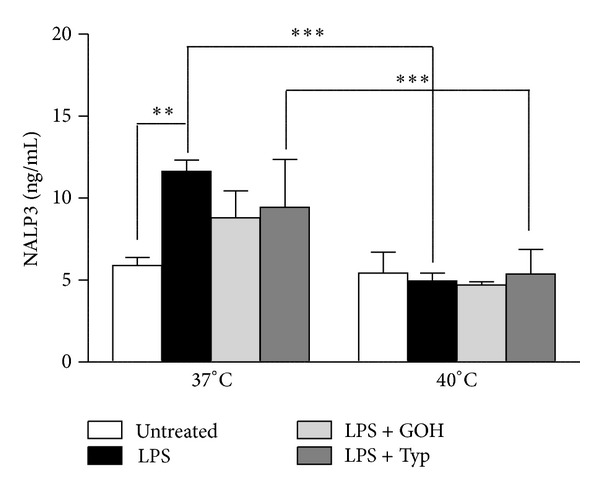
Columns of supernatant level (ng/mL) of NALP3, both at 37°C and at 40°C, in untreated condition and after administration of LPS alone or in combination with GOH or Typ. **P < 0.01; ***P < 0.001; one-way ANOVA test followed by Bonferroni's multiple comparison test. LPS: lipopolysaccharide; GOH: geraniol; Typ: Tipifarnib.

In any treatment condition, levels of NALP3 showed a decreasing trend as the temperature grew. In particular, this trend, showing higher levels at 37°C with respect to 40°C, was statistically significant in the LPS and LPS + GOH groups, while it was less marked in the LPS + Typ group. No differences were observed in the untreated groups (Figure 4).

### 3.5. Apoptosis Was Not Temperature Dependent in MKD Monocytes

**Figure 5:**
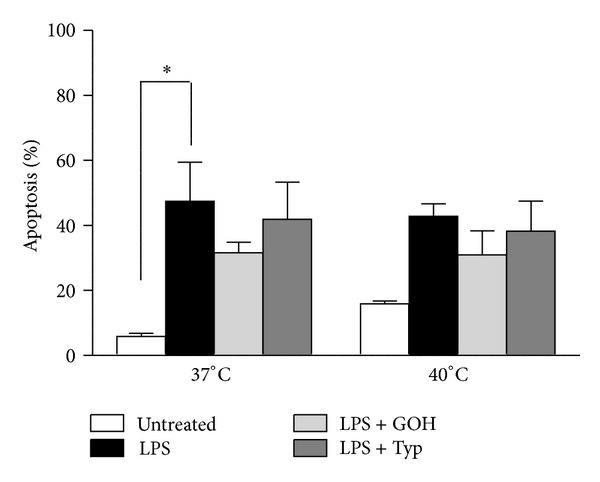
Columns of apoptosis of patients' monocytes both at 37°C and at 40°C, in untreated condition and after administration of LPS alone or in combination with GOH or Typ. *P < 0.05; one-way ANOVA test followed by Bonferroni's multiple comparison test. LPS: lipopolysaccharide; GOH: geraniol; Typ: Tipifarnib.

The percentage of programmed cell death (PCD) significantly increased at 37°C in LPS-treated cells if compared to untreated cells, while drugs treatments slightly reversed this effect. At 40°C we noticed a small increase in PCD in the untreated cells with respect to the same group at 37°C, whereas the other groups showed comparable trends and values with respect to those evaluated at 37°C (Figure 5).

## 4. Discussion

Mevalonate Kinase Deficiency (MKD) is characterized by a proinflammatory predisposition caused by the lack of mevalonate-derived isoprenoids. In MKD, severe inflammatory attacks can arise in response to mild immune stimuli such as stress or vaccinations. Indeed, due to the characteristic irregular recurrence of the attacks in patients, it may be difficult to evaluate the effect of therapeutic interventions. Moreover, studying the effectiveness of a treatment for a rare periodic syndrome is complicated by its frequency variability, disparate severity of symptoms, and its low prevalence: for this reason, a vaccination model has been proposed to evaluate the treatment on demand in a controlled setting [5].

We previously reproduced a model of the disease *in vitro* and *in vivo*, in which shortage of isoprenoids and inflammatory predisposition were obtained by a biochemical block of the pathway, while the inflammatory attack was induced by acute treatment with proinflammatory compounds, such as muramyl dipeptide or LPS, mimicking vaccination [14, 16]. In these models, exogenous isoprenoids such as geraniol or drugs such as Tipifarnib, which are able to partially compensate the metabolic defect, have been shown to slightly correct the abnormal inflammatory predisposition [15]. Before translating these results on clinical trials, we further checked if these therapies maintained their efficacy in “febrile” conditions. In fact, the mevalonate pathway can be modulated by temperature, and it has been suggested that high temperature can worsen the defect in isoprenoid synthesis.

We demonstrated that the LPS treatment versus the untreated condition induces a comparable inflammation response by testing patients' monocytes at 37°C and 40°C: cytokines' profile showed that the temperature does not affect cytokines' levels, acting only on the presence or absence of an inflammatory response.

Furthermore geraniol and Tipifarnib maintain the same efficacy at different temperatures compared to LPS treatment: albeit not statistically significant, the effect of Tipifarnib seemed to be poorly relevant compared to geraniol. This difference in efficacy could be explained considering that GOH and Typ act at different levels of the pathway, respectively, rescuing the lack of intermediate compounds or redirecting the few produced toward an anti-inflammatory way (Figure 1). Unfortunately, although previously studies already demonstrated a synergic effect of these two compounds [15], the small number of monocytes isolated from patients did not allow verifying the potential joint effect of GOH and Typ.

Cytokines following this trend are equally secreted by Th1 (IL-2, INF-*γ*, IL-12, and IL-15), Th2 (IL-4, IL-6), and Th9 (IL-9) cells, thus meaning that the inflammatory phenotype is sustained by both innate and acquired immunity [20]. The inflammatory event arisen after LPS treatment is also driven by different chemokines which act as chemoattractant guiding the migration of immune cells. These molecules can recruit leukocytes, monocytes (VEGF, PDGF-BB, and FGF-basic) neutrophils (IP-10), and other effector cells from the blood [21].

However, in order to induce the inflammatory response, it is important to remark that the action of LPS alone is a necessary but not sufficient condition, since it acts as a trigger for inflammation in a pathological environment [14].

Different considerations should be made on IL-1*β*, the most important inflammatory marker observed in MKD patients, since it seemed to be the only cytokine affected by the temperature. Both at 37°C and at 40°C the LPS treatment induced a statistically significant increase in IL-1*β* values if compared to untreated conditions, but the cytokine's level was dramatically lower at 40°C. This diminishing trend as the temperature grows was observed also after the combined treatment LPS + GOH and, with a significant decrease, after the administration of LPS + Typ.

NALP3 is another molecule that seems to vary as the temperature changes: all the different experimental conditions evaluated were decreased at 40°C with respect to the values at 37°C. These results perfectly relate to the data on IL-1*β*, since NALP3 is the principal molecule involved in the inflammasome processes, and inflammasome activation causes an augmented secretion of IL-1*β* [22, 23].

A fundamental aspect strictly linked with IL-1*β* and NALP3 is the process of apoptosis. This is the final mechanism triggered by NALP3/IL-1*β* driven inflammation. However the percentage of PCD did not significantly change after the same treatments at different temperatures, even if the trend observed was perfectly comparable with that observed for the cytokines (except for IL-1*β*). These results can be exhaustively explained taking into account that the IL-1*β* dependent apoptosis (pyroptosis) is not the only cause of cell death, which instead is principally driven by caspase-3 dependent apoptosis [24–26].

We are aware that due to the small number of patients analyzed we cannot definitively demonstrate the role of temperature and treatments for resetting the inflammatory processes to the homeostatic condition. However we can speculate that the differences in the cytokines' profile and response to the anti-inflammatory drugs (GOH and Tipifarnib) also depend on the physiological interindividual variability of the immune response and the genetic defect of the patient.

Indeed, in MKD residual MK activity varies from <0.5% to 7% depending upon the type of MK mutation [14]. Taking in account that MK activity affects the levels of GGPP and the consequent shortage of geranylgeranylation, monocytes from subjects carrying different MK mutations could need different amounts of exogenous compounds to exhibit the same anti-inflammatory effect. A quantitative residual MK activity evaluation was not performed in our patients since it is not required for clinical definition of the disease.

Unexpectedly, the temperature peak does not correspond to the highest values of proinflammatory markers, so we can speculate that the rescue of the pathway might be caused by the temperature increase itself. This conclusion is supported at a molecular level both by the concentration of released cytokines and by the NALP3-driven inflammasome activation. Thus, temperature represents at the same time not only the acute phase of the disease but also the pathological condition able to trigger the resolution of the phlogistic event.

Based on these findings, we suggest a crucial role of temperature in the modulation of the inflammatory events in MKD patients and suggest strongly considering this variable in future researches aimed at finding a treatment for Mevalonate Kinase Deficiency.

## Figures and Tables

**Table 1:** Background information of patients.

	MKD patients
Case	3
Male/female	3/0
Age (mean ± SD)	11.7 ± 1.2
MKD mutations	V377I/I268T
	V377I/V377I
	G336S/G336S

**Table 2:** Levels of cytokines tested in the untreated and LPS-stimulated MKD patients' monocytes, both at 37°C and 40°C.

Cytokine	Patients' monocytes			
	37°C		40°C	
	Untreated	LPS	Untreated	LPS
IL-1*β*	11.8 ± 10.5	1235 ± 146.2***	2 ± 0.5	494.3 ± 165.8*
IL-1ra	283.8 ± 45.8	864.6 ± 117.4**	267.7 ± 26.4	769.4 ± 47.1**
IL-2	79.9 ± 0.8	114.8 ± 3.3***	70.3 ± 2.1	107.3 ± 5***
IL-4	4.1 ± 0.5	13.8 ± 0.6***	3.8 ± 0.5	12.8 ± 0.9***
IL-5	<2	<2	<2	<2
IL-6	106.4 ± 59.6	21756 ± 3518***	54.4 ± 23.3	19145 ± 2204**
IL-7	<2	<2	<2	<2
IL-8	6047 ± 3068	22571 ± 16271	4852 ± 3581	34676 ± 28376
IL-9	52.1 ± 5.2	197.3 ± 13.3***	42.7 ± 5.6	192.1 ± 8.6***
IL-10	39.8 ± 1	181.9 ± 69.9	37.4 ± 0.9	161.2 ± 42
IL-12(p70)	44.1 ± 2.6	89.3 ± 11**	38.2 ± 1.8	68.9 ± 4.1*
IL-13	4.4 ± 0.1	7.6 ± 1.5	3.6 ± 0.1	6.3 ± 0.2
IL-15	162.2 ± 0.8	185.4 ± 5.3*	153.3 ± 5	176.3 ± 3.2*
IL-17	266.4 ± 0.6	500.3 ± 11.4***	251.6 ± 7.4	495.6 ± 24.5***
Eotaxin	160.5 ± 2.9	216.5 ± 5**	155.9 ± 2.8	205.9 ± 11.2**
FGF-basic	160.6 ± 6.1	262.2 ± 2.2***	163.9 ± 4.4	251.1 ± 10.5***
G-CSF	50.3 ± 2.7	1235 ± 554.8	40.9 ± 2.5	611.1 ± 117.5
GM-CSF	1417 ± 46.6	1718 ± 102.1	1398 ± 17.3	1462 ± 46.9
IFN-*γ*	103.3 ± 7.7	324.3 ± 10.3***	128.2 ± 2.1	308.6 ± 15.7***
IP-10	78.4 ± 4.1	146.2 ± 5.8***	91.7 ± 10.8	131.3 ± 3.2*
MCP-1	519.4 ± 239.2	430 ± 159.7	89.7 ± 32.8	522.7 ± 224.9
MIP-1*α*	28.5 ± 22.3	8980 ± 1899**	19.9 ± 12.9	8234 ± 963.1**
PDGF-BB	80 ± 7.2	129.6 ± 3***	66 ± 3.5	112.7 ± 2.7***
MIP-1*β*	336.1 ± 127.4	33350 ± 3709	343.2 ± 82.7	42701 ± 18032
RANTES	271.9 ± 120.3	474.5 ± 71.9	191.7 ± 70.3	401 ± 50.2
TNF-*α*	66.7 ± 1.9	3613 ± 1076	57.5 ± 6.1	3025 ± 1146
VEGF	77.9 ± 6	116.3 ± 11.5*	68.8 ± 2.5	106.4 ± 5.8*
IL-1*α*	29.6 ± 26	228.2 ± 50.7	4.5 ± 1.6	183.6 ± 70.2
IL-2R*α*	101 ± 7	98.9 ± 19.5	103 ± 15.9	105.1 ± 14.3
IL-3	726.6 ± 182.8	1244 ± 266.9	945.6 ± 113.2	907 ± 246.8
IL-12(p40)	3081 ± 716.6	6938 ± 2344	2754 ± 282.1	3811 ± 1151
IL-16	128.2 ± 47	94.6 ± 14.4	118.3 ± 14.1	130.9 ± 32.9
IL-18	8.3 ± 3.6	12.4 ± 0.9	5.4 ± 2.3	8.4 ± 5.8
CTACK	15.6 ± 11.9	5.3 ± 5.1	24.7 ± 24.5	25.8 ± 21.3
GRO-*α*	2006 ± 903.3	7845 ± 2004	272.1 ± 142.3	5195 ± 996.4
HGF	85.4 ± 3.8	54.2 ± 8.5	56.2 ± 8.5	77.6 ± 10.2
IFN-*α*2	97.8 ± 18.9	78.6 ± 5.7	69.8 ± 15.5	104.2 ± 15.7
LIF	330.2 ± 37.5	302.9 ± 12.3	284.2 ± 17.3	288.3 ± 42.1
MCP-3	455.8 ± 130.6	298.4 ± 32	118.2 ± 64.6	82.5 ± 71.6
M-CSF	127.2 ± 27.3	169.5 ± 31	117.8 ± 7	144.7 ± 10.2
MIF	1681 ± 465.5	1034 ± 190.1	1644 ± 191.1	1913 ± 281.3
MIG	45.6 ± 0.5	37 ± 1.7	36.9 ± 15.3	42.2 ± 3.4
*β*-NGF	<2	<2	<2	<2
SCF	33.5 ± 16.2	<1	<1	5.9 ± 4.9
SCGF-*β*	4375 ± 2343	969.5 ± 930.9	1683 ± 1645	1320 ± 1282
SDF-1*α*	209.4 ± 43.2	425.5 ± 90.8	160.4 ± 19.9	342.9 ± 72.3
TNF-*β*	41.9 ± 2.8	46.7 ± 7.2	42.1 ± 11.2	46.6 ± 11.7
TRAIL	31.6 ± 15.8	35.8 ± 6.5	16.3 ± 15.3	11.4 ± 10.4

Values are expressed as mean ± SEM, in pg/mL; *P < 0.05, **P < 0.01, and ***P < 0.001 versus the untreated groups tested at the same temperature: one-way ANOVA test followed by Bonferroni's multiple comparison test.

**Table 3:** Levels of cytokines tested in the LPS, LPS + GOH, and LPS + Typ treated MKD patients' monocytes, both at 37°C and 40°C.

Cytokine	Patients' monocytes					
	37°C			40°C		
	LPS	LPS + GOH^a^	LPS + Typ^b^	LPS	LPS + GOH^a^	LPS + Typ^b^
IL-1*β*	1235 ± 146.2	657 ± 283.6	1040 ± 73	494.3 ± 165.8	210.8 ± 93.1	343 ± 106.4
IL-1ra	864.6 ± 117.4	632.9 ± 109.6	808.6 ± 100.6	769.4 ± 47.1	610.6 ± 108.5	724.1 ± 34.2
IL-2	114.8 ± 3.3	97.1 ± 9.1	107.2 ± 1.1	107.3 ± 5	92.7 ± 4.1	103.1 ± 6.1
IL-4	13.8 ± 0.6	11.5 ± 1.5	13.6 ± 0.5	12.8 ± 0.9	10.3 ± 1.1	12 ± 1.3
IL-6	21756 ± 3518	17544 ± 6648	20633 ± 3314	19145 ± 2204	12071 ± 4108	18614 ± 2803
IL-9	197.3 ± 13.3	168.8 ± 14.5	190.6 ± 6.5	192.1 ± 8.6	167.7 ± 26.7	181.2 ± 6.5
IL-12(p70)	89.3 ± 11	65 ± 5.8	81.2 ± 4.2	68.9 ± 4.1	70 ± 2	69.3 ± 5.8
IL-15	185.4 ± 5.3	166.3 ± 8.9	177.6 ± 4.6	176.3 ± 3.2	168.8 ± 6.4	176.3 ± 4.4
IL-17	500.3 ± 11.4	427.1 ± 35	478.7 ± 22.4	495.6 ± 24.5	422.7 ± 21.8	465.3 ± 16.4
Eotaxin	216.5 ± 5	186.1 ± 16.6	211.2 ± 3.2	205.9 ± 11.2	187.6 ± 8.2	201 ± 11.6
FGF-basic	262.2 ± 2.2	231.4 ± 13.1	250.2 ± 6.2	251.1 ± 10.5	220.5 ± 8.1	242.2 ± 7.6
IFN-*γ*	324.3 ± 10.3	270.5 ± 34.7	310.6 ± 13.6	308.6 ± 15.7	244.6 ± 26.4	288.8 ± 16.9
IP-10	146.2 ± 5.8	125.7 ± 14.2	145.7 ± 6.4	131.3 ± 3.2	113.8 ± 10.5	129.7 ± 7.6
MIP-1*α*	8980 ± 1899	5308 ± 2213	8306 ± 1780	8234 ± 963.1	4675 ± 1792	7854 ± 1155
PDGF-BB	129.6 ± 3	107.6 ± 8.6	123.2 ± 8.2	112.7 ± 2.7	97.4 ± 11.2	107.9 ± 5.2
VEGF	116.3 ± 11.5	96.4 ± 8.6	115.2 ± 10.1	106.4 ± 5.8	67.8 ± 13.7	112.9 ± 8.8

Values are expressed as mean ± SEM, in pg/mL; ^a^geraniol; ^b^Tipifarnib.
